# A Causal Role of Genetically Elevated Circulating Interleukin-10 in the Development of Digestive Cancers: Evidence from Mendelian Randomization Analysis Based on 29,307 Subjects

**DOI:** 10.1097/MD.0000000000002799

**Published:** 2016-02-18

**Authors:** Wenquan Niu, Qing Pang, Ting Lin, Zhixin Wang, Jingyao Zhang, Minghui Tai, Lingqiang Zhang, Li Zhang, Mingliang Gu, Chang Liu, Kai Qu

**Affiliations:** From the State Key Laboratory of Medical Genomics, Ruijin Hospital, Shanghai Jiaotong University School of Medicine, Shanghai (WN); Department of Hepatobiliary Surgery, The First Affiliated Hospital of Xi’an Jiaotong University, Xi’an, Shaanxi Province (QP, TL, ZW, JZ, MT, LZ, CL, KQ); Department of Hepatobiliary Surgery, The Affiliated Hospital of Qinghai University, Xining, Qinghai (ZW, LZ); Department of Ultrasound Diagnostics, The First Affiliated Hospital of Xi’an Jiaotong University (MT); Department of Ultrasound Diagnostics, Tangdu Hospital, Fourth Military Medical University, Xi’an, Shaanxi Province (LZ); and Chinese Academy of Sciences Key Laboratory of Genome Sciences and Information, Beijing Institute of Genomics (MG), Chinese Academy of Sciences, Beijing, China.

## Abstract

Recent studies have observed a high level of circulating interleukin-10 (IL-10) in patients with digestive cancers, yet whether elevated IL-10 is causally associated with digestive cancers so far remained unresolved.

We therefore meta-analyzed available observational studies with Mendelian randomization method to explore this causal association by employing IL-10 gene 3 variants (-592C>A, -819C>T, and -1082A>G) as instruments.

Data were available from 52 articles encompassing 29,307 subjects. Subgroup analysis by cancer type indicated that -1082A>G was associated with increased risk of gastric cancer (odds ratio [OR] = 1.19; 95% confidence interval [CI]: 1.05–1.35; *P* = 0.006), and the association was reinforced for intestinal type gastric cancer (OR = 1.26; 95%CI: 1.09–1.44; *P* = 0.001). By ethnicity, risk estimate for -1082G allele carriers was increased by 21% for digestive cancers in East Asians (95%CI: 1.05–1.40; *P* = 0.009). As for the genotype–phenotype association, carriers of -1082G allele had an overall 20.21 pg/mL higher IL-10 level than those with -1082AA genotype (*P* = 0.023). In further Mendelian randomization analysis, the predicted OR for 10 pg/mL increment in IL-10 was 1.14 (95%CI: 1.01–16.99) in gastric cancer.

Our findings provided evidence for a causal role of genetically elevated IL-10 in the development of gastric cancer, especially in East Asians and for intestinal type gastric cancer.

## Abstract 2

Supplemental Digital Content is available in the text

## INTRODUCTION

A constellation of malignancies that originate from digestive organs, such as stomach, colon, and liver, constitutes digestive cancers. In many patients with digestive system malignancies, there is a strong inherited predisposition with epidemiological data generated from human twin and family studies. For example, family members who have a mutation in a mismatch repair gene are observed to have a much higher rate of colorectal cancer than those who do not have the mutation.^1^ Chiba et al^2^ have written an excellent review on the genetic underpinnings of digestive cancers and highlighted that genes encoding inflammatory factors, interleukin family members in particular, play a contributory role in the pathogenesis of various cancers, particularly in digestive organs.

One of the most intensively evaluated members in interleukin family is interleukin-10 (IL-10), a key cytokine involved in immune response and carcinogenesis. Recent studies have observed a high level of circulating IL-10 in patients with digestive cancers and its association with poor prognosis.^3^ Accumulating evidence suggested that interindividual variation in circulating IL-10 may arise from common polymorphic variation in IL-10 gene. The genomic sequence of IL-10 gene is highly polymorphic and 3 promoter variants, viz -592C>A (rs1800872), -819C>T (rs1800871), and -1082A>G (rs1800896), in possible association with alterations of IL-10 function are well-defined from different populations with varying prevalence.^4,5^ It is reasonable to expect that if IL-10 is involved in the underlying pathological process of digestive cancers, the inherited genetic determinants that alter circulating IL-10 should affect cancer risk in the direction and magnitude predicted by its circulating level.

As available evidence regarding the association between circulating IL-10 and digestive cancers is mainly derived from observational studies, it is difficult to disentangle causation from association, especially in the presence of confounding. Mendelian randomization is considered as a viable method to obtain the causality of an exposure-disease association using genetic determinants.^6,7^ Bearing this in mind, we first meta-analyzed the association of 3 aforementioned variants in IL-10 gene with digestive cancer risk and changes of circulating IL-10 level. Second, we selected the variant(s) that can be simultaneously predictive of digestive cancers and circulating IL-10 as an instrument to explore their potential causal relevance by implementing Mendelian randomization method.

## METHODS

According to the preferred reporting items for systematic reviews and meta-analyses (PRISMA) statement, this meta-analysis was carried out (Supplementary PRISMA checklist).^8^ Our research (meta-analysis) is not needed to be approved by the ethics committee according to PRISMA guidelines.

### Search Strategy

A literature search for observational studies that investigated the association between IL-10 gene 3 variants (-592C>A, -819C>T, and -1082A>G) and all types of digestive cancers was conducted of PubMed and Google Scholar databases covering the period from the earliest possible year to May 1, 2015. Subject terms used for the search included “interleukin 10,” “interleukin-10,” “IL 10,” “IL-10,” “gastric or stomach,” “colorectal or colon or rectal,” “esophageal,” “liver or hepatic or hepatocellular,” “pancreatic,” “gallbladder or biliary,” “cancer or carcinoma or tumor or sarcoma or leiomyoma,” together with “polymorphism or genetic or variant or mutation or allele or genotype.” Citations in retrieved articles as well as reviews on the same topic were also searched where relevant. Only articles published in English language were identified.

### Trial Selection

Two investigators (KQ and WN) independently scanned the titles and abstracts to evaluate their eligibility. The full text was reviewed when an article cannot be rejected based on its title or abstract. If more than one article from the same study group or the same cohort, we only extracted the data from the most recent or complete articles.

### Inclusion/Exclusion Criteria

The following criteria were used for the literature selection in this meta-analysis: clinical endpoints should be digestive cancers including esophageal cancer, gastric cancer, colorectal cancer, hepatocellular carcinoma, biliary tract cancer, and pancreatic cancer; studies should follow either a retrospective or a prospective case–control design; and the genotype/allele counts of at least 1 of the 3 variants examined and/or circulating IL-10 level across genotypes of either variant should be provided. Studies were excluded (1 point was sufficient for exclusion) if they investigated the gene function, disease progression, severity and the response to treatment, or survival. Additionally, conference abstracts or proceedings, case reports or series, editorials, narrative reviews, meta-analyses, and the non-English articles were also excluded.

### Data Extraction

Two investigators (KQ and WN) independently extracted data using a standardized excel template. Disagreements were resolved by a 3rd investigator (CL). Data were collected on the 1st author, publication year, ethnicity of the study subjects, cancer type, study design, sample size, the genotype/allele counts of 3 examined variants between cases and controls, and the characteristics of the study subjects, if available, including age, gender, body mass index (BMI), smoking, drinking, family history of cancers, history of digestive diseases, and bacteria or virus infection status.

### Statistics

In this meta-analysis, the association of 3 variants in IL-10 gene with digestive cancer risk was calculated under 3 genetic models of inheritance, including allelic model, homozygous genotypic model, and dominant model. Weighted odds ratios (ORs) and the corresponding 95% confidence intervals (95% CIs) were quantified by a random-effects model.^9^ Heterogeneity between studies was calculated by the inconsistency index (*I*^2^) statistic and *I*^2^ > 50% was designated as a threshold to indicate significant heterogeneity. Publication bias was assessed by Begg funnel plot and the corresponding Egger test. A 10% level of significance for the Egger test was considered as the presence of publication bias.^10^

Predefined subgroup analyses were performed a priori according to cancer type (esophageal cancer, gastric cancer, colorectal cancer, hepatocellular carcinoma, biliary tract cancer, or pancreatic cancer), ethnicity of the study subjects (Caucasian, East Asian, Latinos, or mixed), source of controls (population-based or hospital-based), study design (prospective or retrospective), and total sample size (<500 subjects or ≥500 subjects). For gastric cancer, further subgroup analyses were undertaken according to its anatomic type (gastric cardia or noncardia cancer) and histologic type (diffuse or intestinal type). Additionally, to account for the sources of heterogeneity from continuous confounders such as age, sex, BMI, smoking, drinking, family history of cancer, and *Helicobacter pylori* infection (only for gastric cancer studies), a set of meta-regression analyses were performed to evaluate the association of 3 examined variants with digestive cancer risk. For the variant that was simultaneously associated with the significant risk of digestive cancers or its subtypes and the significant changes of circulating IL-10 level, Mendelian randomization analysis was accordingly performed.

Data management and statistical analyses described above were completed with the STATA software (StataCorp, TX, version 11.2).

## RESULTS

### Eligible Articles

**FIGURE 1:**
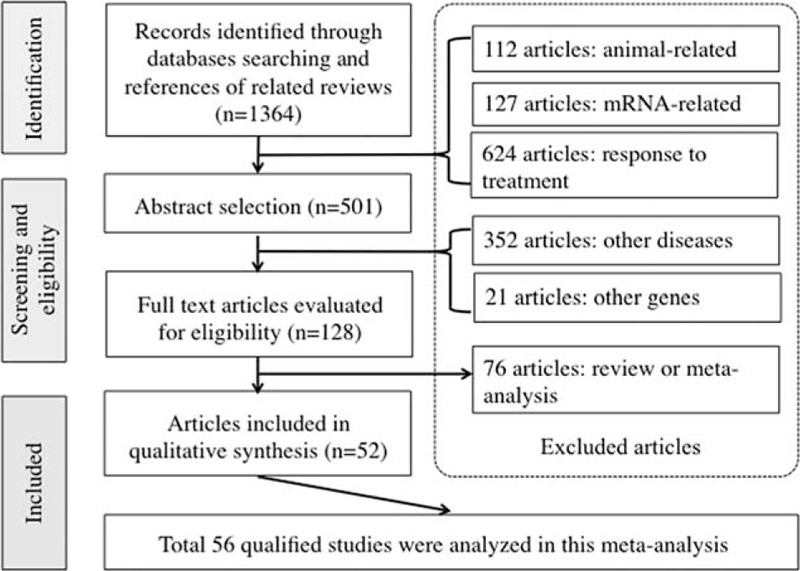
Flow diagram of search strategy and study selection.

Figure 1 is a flow diagram that schematizes the process of article exclusion with specific reasons. Altogether 128 potentially relevant articles were identified according to the search strategy, and 52 of them were qualified after applying further the inclusion/exclusion criteria. All 52 qualified articles were written in English and published between 2003 and 2014.^11–62^ As data within 3 articles were separately provided by cancer type, the final analysis included a total of 56 independent studies (Table 1).

**TABLE 1:**
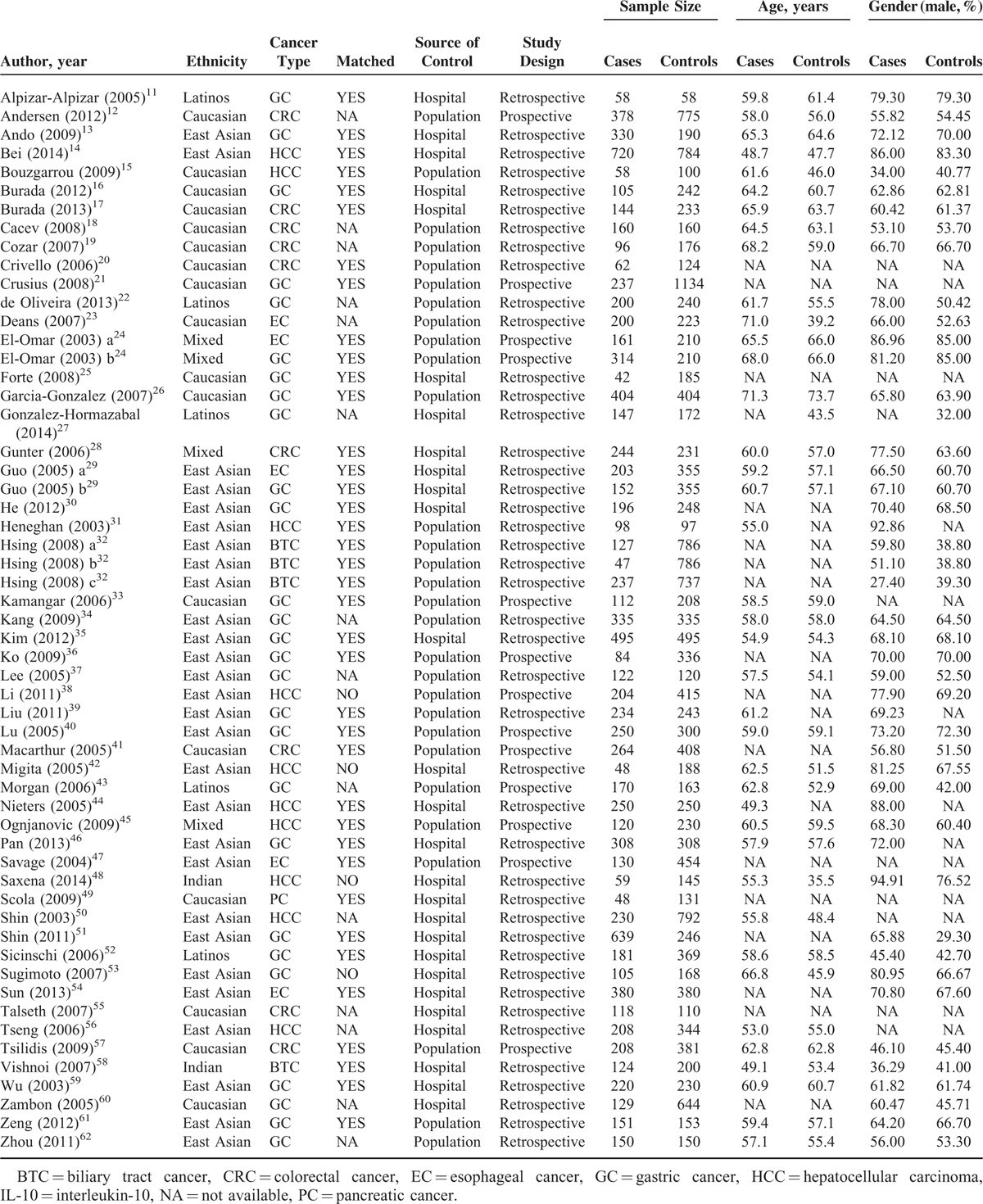
Baseline Characteristics of Eligible Studies for Association of IL-10 Gene 3 Variants With Digestive Cancers

### Study Characteristics

The baseline characteristics of all 56 qualified studies are shown in Table 1, and the genotype distributions and allele frequencies of 3 examined variants (-592C>A, -819C>T, and -1082A>G) in IL-10 gene between cases and controls are provided in Supplementary Table 1. In this meta-analysis, 27 studies were conducted for gastric cancer, 10 for hepatocellular carcinoma, 9 for colorectal cancer, 5 for esophageal cancer, 4 for biliary tract cancer, and 1 for pancreatic cancer. There were 30 studies involving Asians, 17 involving Caucasians, 5 involving Latinos, and 4 involving the mixed populations. Twenty-nine studies were conducted on a population-based design and 27 were on a hospital-based design. Thirty (53.57%) of 56 qualified studies had total sample size equal to or more than 500 subjects.

### Association of IL-10 Gene 3 Variants With Digestive Cancers

**FIGURE 2:**
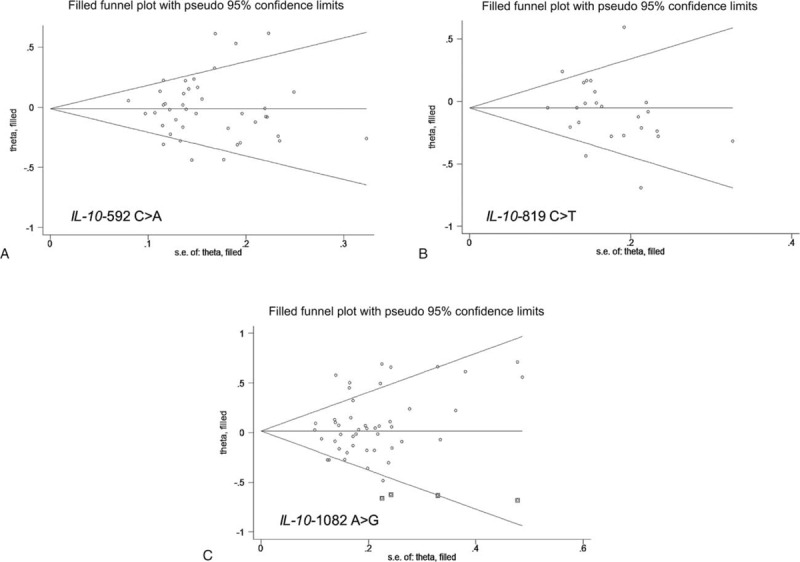
Funnel plots for studies investigating the effect of IL-10 gene 3 variants on digestive cancer risk. Vertical axis represents the log of OR; horizontal axis represents the SE of log (OR). Funnel plots are drawn with 95% confidence limits. The graphic symbols represents the data in the plot be sized proportional to the inverse variance. IL-10 = interleukin-10, OR = odds ratio, SE = standard error.

Pooling 56 qualified studies together revealed no significant association between 3 examined variants (-592C>A, -819C>T, and -1082A>G) and digestive cancers under all 3 genetic models, yet there was evident heterogeneity. There were low probabilities of publication bias as reflected by the suggestive symmetry of Begg funnel plots (Figure 2), as well as it is associated Egger tests (*P* = 0.34, 0.38, and 0.07 for allelic, homozygous genotypic, and dominant models, respectively).

What is more, for -592C>A and -819C>T, there was no indication of significance in subgroup analyses, except for a relatively weak association between -592C>A and biliary tract cancer (OR = 1.30; 95% CI: 1.03–1.63; *P* = 0.028) and between -819C>T and gastric cancer (OR = 0.87; 95% CI: 0.77–0.97; *P* = 0.016) under allelic model (Table S2).

**FIGURE 3:**
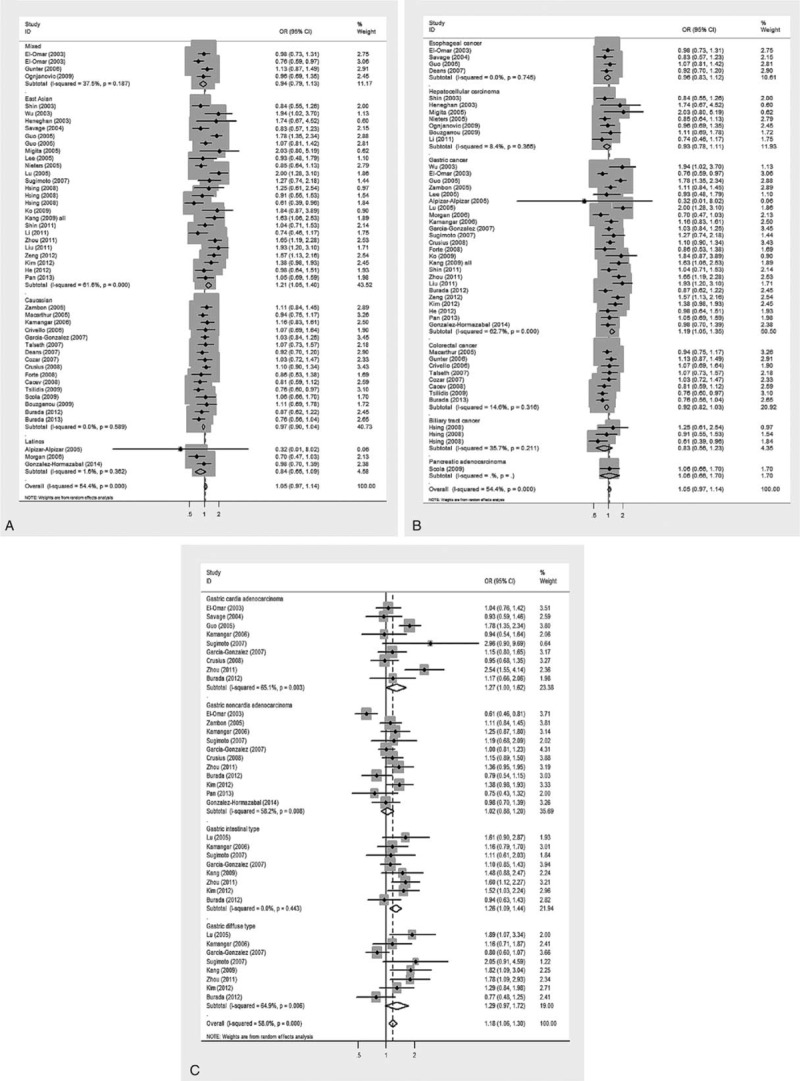
Risk estimates of IL-10 gene -1082A>G for cancer risk under the allelic model. (A) East Asian population, (B) gastric cancer groups, and (C) intestinal type of gastric cancer. The summary treatment effect (OR) is shown by the middle of a solid diamond whose left and right extremes represent the corresponding 95% CI. Horizontal axis represents OR values, which were calculated against healthy controls. 95% CI = 95% confidence interval, IL-10 = interleukin-10, OR = odds ratio.

To account for the potential sources of between-study heterogeneity, a set of predefined subgroup analyses were conducted for -1082A>G (Table 2). By ethnicity, significant association between -1082G allele and digestive cancer risk was observed in East Asians under allelic (OR = 1.21; 95% CI: 1.05–1.40; *P* = 0.009; Figure 3A), homozygous genotypic (OR = 1.86; 95% CI: 1.26–2.76; *P* = 0.002), and dominant (OR = 1.22; 95% CI: 1.04–1.43; *P* = 0.013) models (Supplementary Figure 1). By cancer type, risk estimates of -1082A>G were significant for gastric cancer under allelic (OR = 1.19; 95% CI: 1.05–1.35; *P* = 0.006; Figure 3B), homozygous genotypic (OR = 1.48; 95% CI: 1.09–2.02; *P* = 0.013), and dominant (OR = 1.21; 95% CI: 1.04–1.41; *P* = 0.012) models (Supplementary Figure 2) for colorectal cancer under only dominant model (OR = 0.84; 95% CI: 0.72–0.99; *P* = 0.034). The heterogeneity between studies was relatively low for all cancer types except gastric cancer. Further subgroup analyses by anatomic types or histologic types of gastric cancer showed that risk estimates of -1082A>G were strongly reinforced for intestinal type of gastric cancer under allelic (OR = 1.26; 95% CI: 1.09–1.44; *P* = 0.001; Figure 3C), homozygous genotypic (OR = 1.42; 95% CI: 1.04–1.94; *P* = 0.028), and dominant (OR = 1.33; 95% CI: 1.11–1.60; *P* = 0.002) models (Supplementary Figure 3), yet without observable heterogeneity (*I*^2^ = 0.0%) and this estimate was only significant for gastric cardia cancer under dominant model (OR = 1.34; 95% CI: 1.04–1.71; *P* = 0.021).

**TABLE 2:**
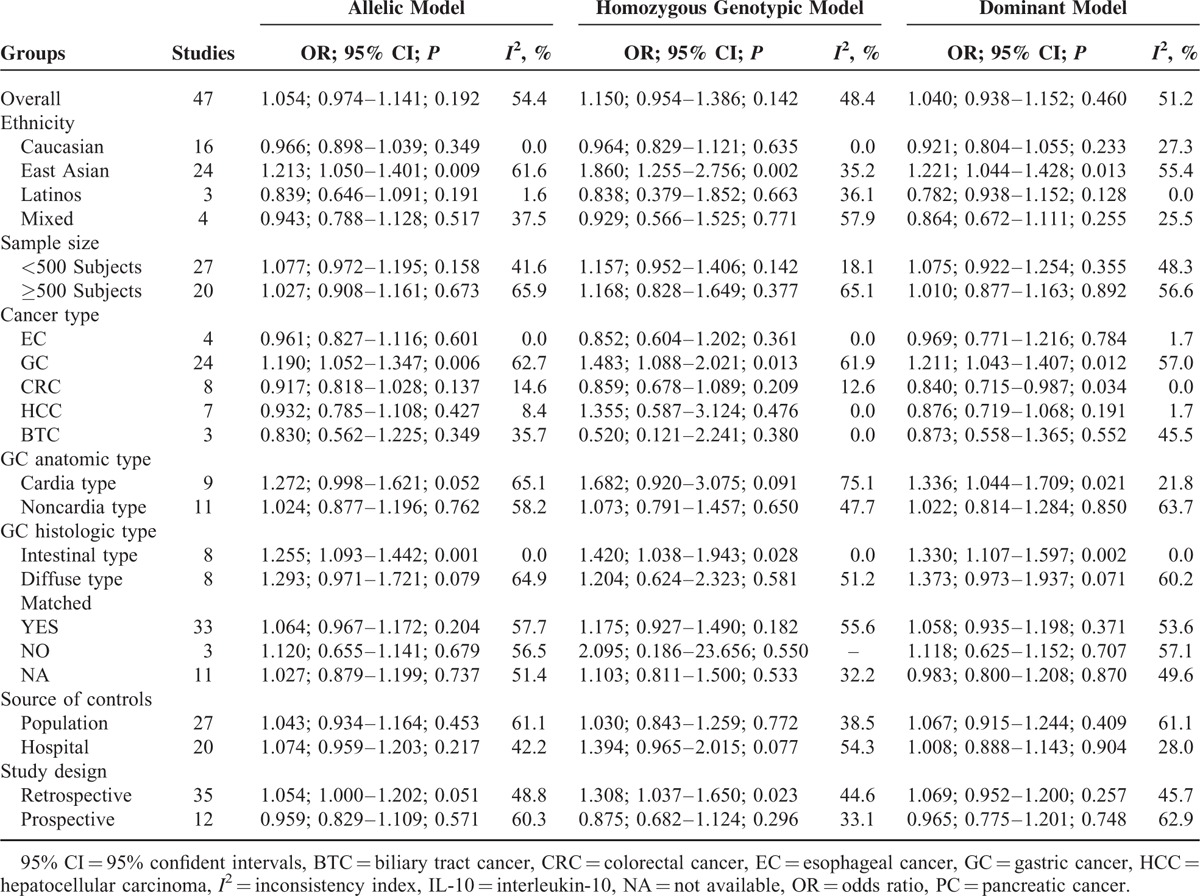
Overall and Subgroup Analyses of IL-10 Gene -1082A>G With Digestive Cancer Risk

By study design or sample size, there were no significant differences in pooled risk estimates between the population- and hospital-based studies and between small and large studies.

### Meta-Regression Analysis

**FIGURE 4:**
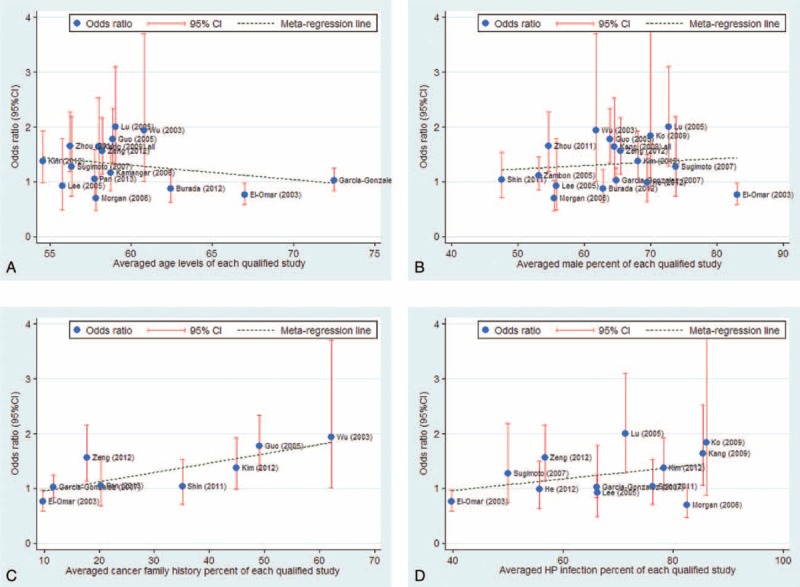
Meta-regression fitted lines by age (A), sex (B), family history of cancer percent (C), and *Helicobacter pylori* infection percent (D).

To explore additional sources of between-study heterogeneity in gastric cancer, a univariate meta-regression model was constructed that included age, sex, BMI, smoking, drinking, family history of cancer, and *Helicobacter pylori* infection as independent variables. Age, sex, family history of cancer, and *Helicobacter pylori* infection were observed to significantly affect the relationship between -1082A>G and gastric cancer susceptibility (*P* = 0.048, 0.028, 0.014, and 0.018, respectively; Figure 4). In contrast, BMI, smoking, and drinking were not observed to affect the relationship between -1082A>G and gastric cancer susceptibility.

### Association of IL-10 Variants With Circulating IL-10 Level

**FIGURE 5:**
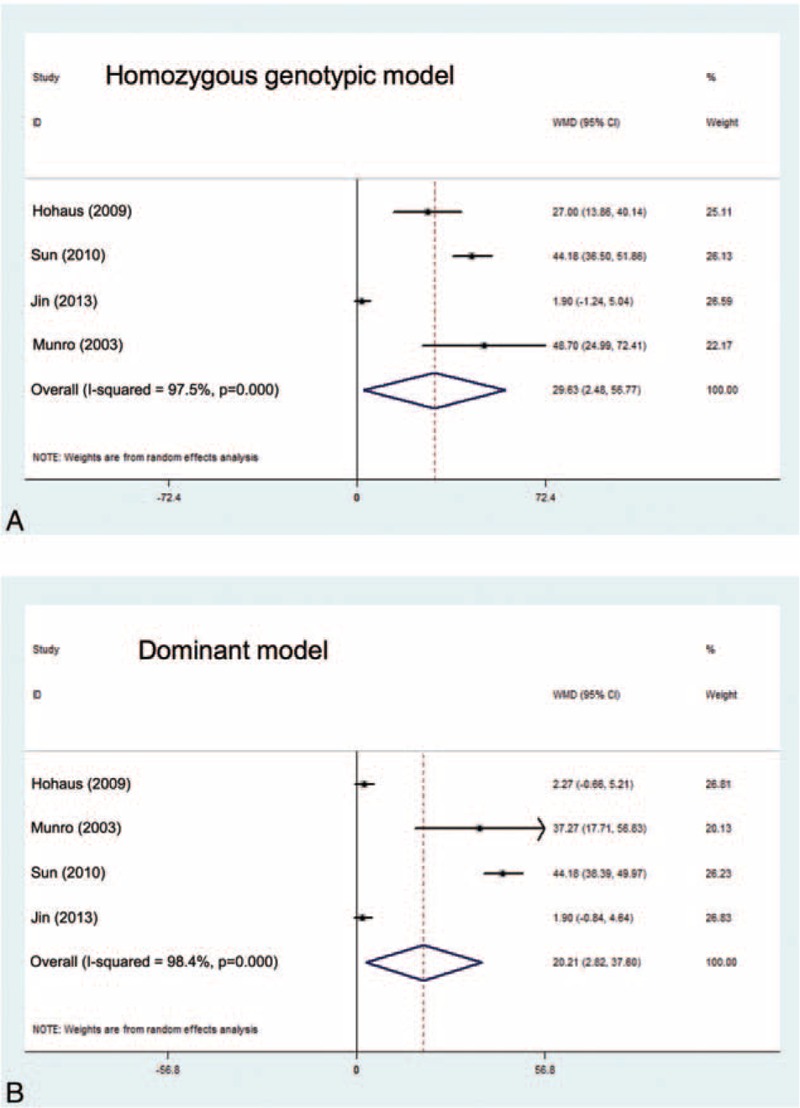
Comparison of IL-10 gene -1082A>G genotypes for circulating IL-10 level in the allelic (left pane) and dominant (right pane) models. The summary treatment effect (SMD) is shown by the middle of a solid diamond whose left and right extremes represent the corresponding 95% CI. 95% CI = 95% confidence interval, IL-10 = interleukin-10, SMD = standard mean difference.

Genotype–phenotype association was based on 10 studies with circulating IL-10 level measured in cancer patients or healthy controls. Eight out of 10 studies were conducted -592C>A and -819C>T, and 4 for -1082A>G (Supplementary Table 3). Circulating IL-10 level was significantly elevated in -1082G allele carriers under homozygous genotypic (standard mean difference [SMD] = 29.63 pg/mL; 95% CI: 2.48–56.77; *P* = 0.032) and dominant (SMD = 20.21 pg/mL; 95% CI: 2.82–37.60; *P* = 0.023) models (Figure 5). There were low probabilities of publication bias for both models as reflected by the Begg funnel plots and the Egger tests (*P* = 0.31 and 0.32, respectively). As expected, there were no significant differences in the changes of circulating IL-10 level for -592C>A and -819C>T under both models (Supplementary Figure 4).

### Prediction of Circulating IL-10 for Gastric Cancer by Mendelian Randomization

Under the assumptions required for Mendelian randomization and assuming a linear-logistic relationship between difference of circulating IL-10 level and odds of gastric cancer, the predicted ORs for 5 and 10 pg/mL IL-10 increment were 1.07 (95% CI: 1.01–4.12) and 1.14 (95% CI: 1.01–16.99), respectively. Since the 95% CIs of both predicted estimates excluded the null hypothesis value of 1, it is safe to reject the null hypothesis at a 5% significance level, and suggested a potentially causal association of circulating IL-10 level with gastric cancer.

## DISCUSSION

By meta-analyzing of the data from 56 studies and on 29,307 subjects, we investigated 3 promoter variants (-592C>A, -819C>T, and -1082A>G) in IL-10 gene and its circulating level in association with the risk of digestive cancers. The most noteworthy finding of this study was that genetically elevated circulating IL-10 was significantly associated with an increased risk of gastric cancer by employing -1082A>G as an instrument. Moreover, extending previous understandings of the close relationship between IL-10 genetic variants and gastric cancer, we further pinpointed a remarkable contribution of -1082A>G to intestinal type gastric cancer. To the best of our knowledge, this is the first meta-analysis interrogating the causal relevance of circulating IL-10 and gastric cancer by implementing Mendelian randomization method.

Compared with other cancers, digestive cancers are more vulnerable to the impact of chronic inflammation, as digestive organs expose a large internal surface area to external environments. Besides exposing to chemical or biological agents of ingested foods, these organs also provide a place for many microorganisms, leading to the infiltration of many immunocytes and cytokines in pathologic conditions.^2^ There is growing recognition that IL-10 plays a key role in maintaining intestinal immune homeostasis,^63,64^ and indeed high circulating IL-10 level was observed in a large proportion of digestive cancer patients with poor prognosis, especially for gastric cancer,^3^ colorectal cancer,^65^ and hepatocellular carcinoma.^66^ In addition, accumulating evidence from functional investigations suggested that upregulation of IL-10 production from immunocytes exerted a great impact in tumor growth, immigration, and immune surveillance.^67,68^ However, whether circulating IL-10 is simply a biomarker for digestive cancers or whether elevated IL-10 level actually contributes directly to carcinogenesis is currently unclear. With this in mind, we employed the classical Mendelian randomization method in this meta-analysis and found that patients with 10 pg/mL increment in circulating IL-10 were 1.14 times more likely to develop gastric cancer. Nevertheless, a note of caution should be sounded in the interpretation of this finding, considering the unstable nature of IL-10 in the circulation according to a previous report (plasma half-life of IL-10 ranges from 2.7 to 4.5 hours),^69^ calling for a robust validation from well-designed, large studies with multiple measurements in circulating IL-10 to quantify this effect size reliably.

The transcriptional activation and protein production of a cytokine gene depends on the binding of regulatory factors to specific recognition sequences in the promoter. Mutations in promoter sequences of some cytokine genes may alter transcription factor recognition sites and consequently affect cytokine production.^5^ Previous studies have reported that a number of putative recognition sites are present in the IL-I0 promoter, such as PEA1, API, and an ETS-like element. The IL-10 variant (-1082A>G) that investigated in the present study lies within an ETS-like recognition site^70^ and may consequently affect the binding of this transcription factor and influence IL-10 production.

According to the Lauren classification, gastric cancer can be classified into adenocarcinomas of the diffuse and the intestinal type, and the latter is believed to arise secondary to chronic gastritis and be associated with relatively better prognosis.^71^ Although enormous efforts have been made to explore genetic susceptibility of IL-10 gene -1082A>G to gastric cancer risk,^72–74^ there has been little attention on specific subtypes of gastric cancer. Ni et al^73^ once performed a pilot analysis on 4 studies and found a weak association between -1082G allele and intestinal type gastric cancer. Via a comprehensive meta-analysis, we, in subgroup analyses, confirmed the contributory role of -1082A>G in the pathogenesis of intestinal type gastric cancer, and this role is less likely biased by between-study heterogeneity. Going forward, it will be of clinical importance using -1082A>G to refine risk stratification and identify high-risk individuals for cost-effective screening, surveillance, and early detection of intestinal type gastric cancer.

Despite the clear advantages of this meta-analysis including the detailed spectrum of digestive cancers, the implementation of Mendelian randomization and the large sample sizes, several possible limitations should be noted. First, we only examined 3 promoter variants in IL-10 gene, and investigation on other variants in or flanking IL-10 gene, especially some low-penetrance genes will be encouraged. It seems likely that -1082A>G by itself makes only a small or moderate contribution to risk prediction for gastric cancer patients, but whether this variant integrated with other risk factors will enhance prediction requires additional research. Second, a pleiotropic impact of the instrumental variant -1082A>G, a major drawback of Mendelian randomization, used in this meta-analysis, could not be totally excluded due to insufficient data in qualified studies. Third, nearly all involved studies in this meta-analysis had circulating IL-10 measured only once and did not reflect its long-term level in the development of digestive cancers. Therefore, the jury must refrain from drawing a conclusion until large, well-performed studies confirm or refuse our findings.

## CONCLUSION

Taken together, our findings provided evidence for a causal role of genetically elevated circulating IL-10 in the development of gastric cancer by employing IL-10 gene -1082A>G as an instrument, and the risk association of this variant with digestive cancers was more evident in patients with intestinal type gastric cancer. These findings warrant further studies to investigate the exact mechanisms of circulating IL-10 level in the development of gastric cancer.

## Supplementary Material

Supplemental Digital Content

## Supplementary Material

Supplemental Digital Content
