# Supplementary material for: A Causal Role of Genetically Elevated Circulating Interleukin-10 in the Development of Digestive Cancers: Evidence from Mendelian Randomization Analysis Based on 29,307 Subjects
Source: Medicine (Baltimore). 2016 Feb 18;95(7):e2799. doi: 10.1097/MD.0000000000002799 (PMC4998630; doi:10.1097/MD.0000000000002799)
Supplement: Supplemental Digital Content [file medi-95-e2799-s002.pdf]

Supplementary figure legend

Figure S1. Risk estimates of IL-10 gene -1082A>G for cancer risk in East Asian population under the homozygous genotypic (A) and dominant (B) models. The summary treatment effect (OR) is shown by the middle of a solid diamond whose left and right extremes represent the corresponding 95% confidence interval (95% CI). Horizontal axis represents OR values, which were calculated against healthy controls.

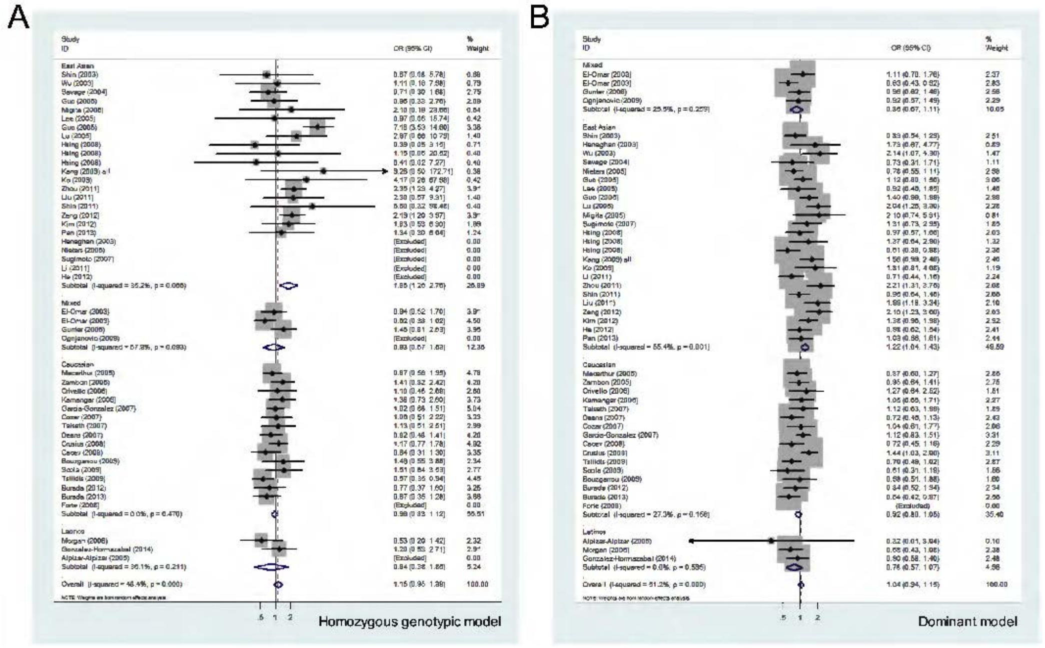

Figure S2. Risk estimates of IL-10 gene -1082A>G for gastric cancer under the homozygous genotypic (A) and dominant (B) models. The summary treatment effect (OR) is shown by the middle of a solid diamond whose left and right extremes represent the corresponding 95% confidence interval (95% CI). Horizontal axis represents OR values, which were calculated against healthy controls.

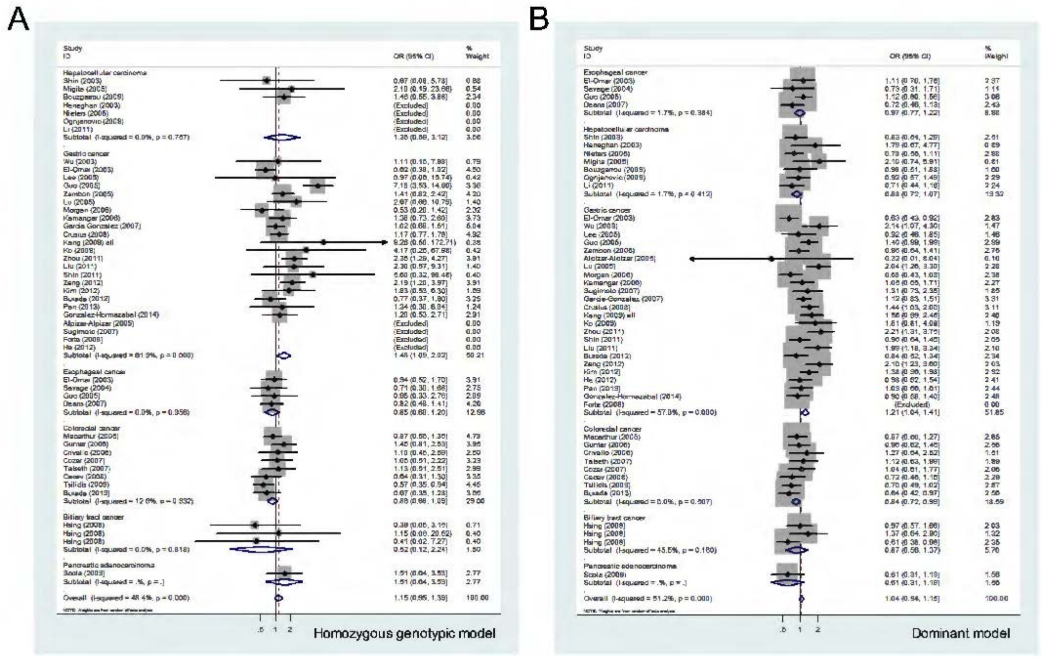

Figure S3. Risk estimates of IL-10 gene -1082A>G for intestinal type of gastric cancer under the homozygous genotypic (A) and dominant (B) models. The summary treatment effect (OR) is shown by the middle of a solid diamond whose left and right extremes represent the corresponding 95% confidence interval (95% CI). Horizontal axis represents OR values, which were calculated against healthy controls.

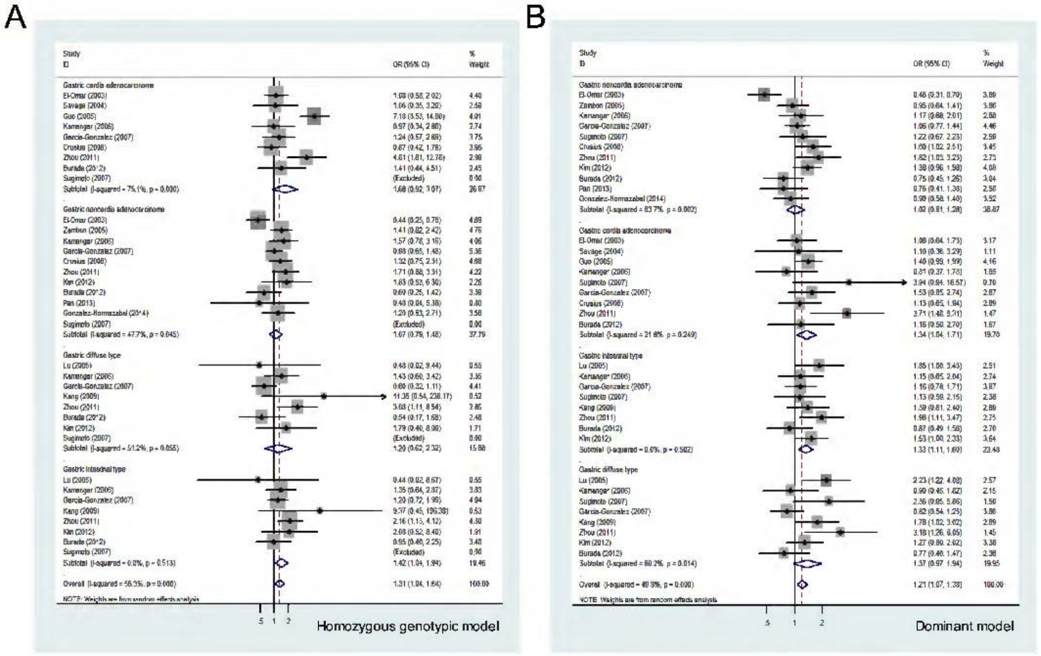

Figure S4. Comparison of IL-10 gene -592 C>A or -819 C>T genotypes for circulating IL-10 level in the allelic and dominant models. A. -592 C>A genotypes under allelic model; B. -592 C>A genotypes under dominant model; C. -819 C>T genotypes under allelic model; D. -819 C>T genotypes under dominant model. The summary treatment effect (standard mean difference or SMD) is shown by the middle of a solid diamond whose left and right extremes represent the corresponding 95% confidence interval (95% CI).

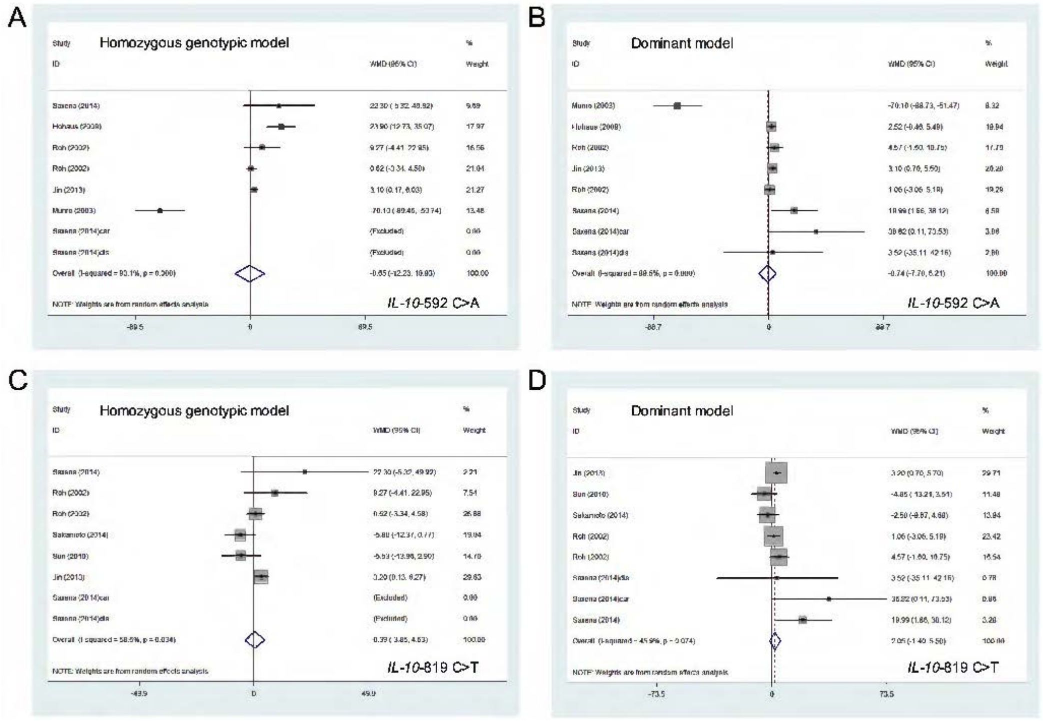

Supplementary Table S1. The genotype distributions of three examined variants in IL-10 gene between digestive cancer patients and controls in all qualified studies

Author (year)	IL-10 gene -592 C>A (rs1800872)						IL-10 gene -819C>T (rs1800871)						IL-10 gene -1082A>G (rs1800896)					
	Case_CC	Case_CA	Case_AA	Control_CC	Control_CA	Control_AA	Case_CC	Case_CT	Case_TT	Control_CC	Control_CT	Control_TT	Case_AA	Case_AG	Case_GG	Control_AA	Control_AG	Control_GG
Alpizar-Alpizar (2005)	21	20	3	18	21	5	25	16	4	18	24	3	45	0	0	43	1	0
Andersen (2012)	238	116	24	470	261	44	NA	NA	NA	NA	NA	NA	NA	NA	NA	NA	NA	NA
Ando (2009)	23	147	160	16	97	77	NA	NA	NA	NA	NA	NA	NA	NA	NA	NA	NA	NA
Bei (2014)	52	312	356	79	313	392	NA	NA	NA	NA	NA	NA	NA	NA	NA	NA	NA	NA
Bouzgarrou (2009)	NA	NA	NA	NA	NA	NA	NA	NA	NA	NA	NA	NA	24	24	10	42	49	12
Burada (2012)	NA	NA	NA	NA	NA	NA	NA	NA	NA	NA	NA	NA	43	49	13	89	118	35
Burada (2013)	NA	NA	NA	NA	NA	NA	NA	NA	NA	NA	NA	NA	65	60	19	80	118	35
Cacev (2008)	83	64	13	104	52	4	83	64	13	106	51	3	54	86	20	43	92	25
Cozar (2007)	52	41	2	98	63	14	52	41	2	98	63	14	31	48	17	58	87	30
Crivello (2006)	31	28	3	69	48	7	NA	NA	NA	NA	NA	NA	16	34	12	38	60	26
Crusius (2008)	148	78	11	642	397	83	145	72	12	636	378	80	54	131	50	340	526	268
de Oliveira (2013)	100	96	4	169	65	6	NA	NA	NA	NA	NA	NA	NA	NA	NA	NA	NA	NA
Deans (2007)	NA	NA	NA	NA	NA	NA	NA	NA	NA	NA	NA	NA	53	93	54	46	120	57
El-Omar (2003) a	107	44	10	127	70	13	NA	NA	NA	NA	NA	NA	42	87	32	59	103	48
El-Omar (2003) b	178	101	35	127	70	13	NA	NA	NA	NA	NA	NA	120	133	61	59	103	48
Forte (2008)	NA	NA	NA	NA	NA	NA	NA	NA	NA	NA	NA	NA	0	37	5	0	149	36
Garcia-Gonzalez (2007)	237	143	24	245	131	28	NA	NA	NA	NA	NA	NA	123	204	77	133	189	82
Gonzalez-Hormazabal (2014)	55	73	19	78	83	11	NA	NA	NA	NA	NA	NA	79	54	14	88	71	13
Gunter (2006)	NA	NA	NA	NA	NA	NA	125	76	21	117	79	13	61	114	47	55	123	29
Guo (2005) a	NA	NA	NA	NA	NA	NA	NA	NA	NA	NA	NA	NA	117	81	5	267	164	12
Guo (2005) b	NA	NA	NA	NA	NA	NA	NA	NA	NA	NA	NA	NA	93	56	30	267	164	12
He (2012)	18	96	82	28	128	92	18	96	82	28	128	92	154	42	0	194	54	0
Heneghan (2003)	11	38	49	13	30	54	11	38	49	13	30	54	86	12	0	90	7	0
Hsing (2008) a	52	16	57	352	90	340	17	52	55	90	353	334	107	18	0	664	108	7
Hsing (2008) b	6	21	19	352	90	340	21	6	20	90	353	334	38	9	0	664	108	7
Hsing (2008) c	23	91	121	82	334	318	23	92	122	82	335	311	231	23	1	624	99	7
Kamangar (2006)	68	38	6	109	82	17	58	35	5	80	62	10	38	47	27	72	96	37
Kang (2009)	34	157	142	41	145	146	NA	NA	NA	NA	NA	NA	281	49	4	289	35	0
Kim (2012)	50	214	231	56	191	248	50	214	231	56	191	248	416	72	7	435	56	4
Ko (2009)	0	33	25	0	110	121	0	33	25	0	111	122	46	9	1	192	22	1
Lee (2005)	8	62	52	7	60	53	NA	NA	NA	NA	NA	NA	104	17	1	101	18	1
Li (2011)	16	134	0	34	313	0	22	144	0	39	323	0	132	26	0	278	77	0
Liu (2011)	39	96	99	28	106	109	39	96	99	28	106	109	189	39	6	217	23	3
Lu (2005)	NA	NA	NA	NA	NA	NA	NA	NA	NA	NA	NA	NA	201	43	6	268	29	3
Macarthur (2005)	151	99	8	248	133	22	NA	NA	NA	NA	NA	NA	61	125	71	86	202	115
Migita (2005)	8	23	17	25	78	85	8	23	17	25	78	85	42	5	1	176	10	2
Morgan (2006)	NA	NA	NA	NA	NA	NA	NA	NA	NA	NA	NA	NA	121	42	7	101	49	11
Nieters (2005)	NA	NA	NA	NA	NA	NA	0	119	130	0	135	115	130	119	0	115	135	0
Ognjanovic (2009)	NA	NA	NA	NA	NA	NA	NA	NA	NA	NA	NA	NA	39	79	0	67	147	0
Pan (2013)	36	128	144	31	135	142	NA	NA	NA	NA	NA	NA	263	41	4	264	41	3
Savage (2004)	51	51	17	171	166	49	17	46	53	49	163	170	8	26	81	20	81	284
Saxena (2014)	9	47	3	36	87	22	9	47	3	36	87	22	NA	NA	NA	NA	NA	NA
Scola (2009)	NA	NA	NA	NA	NA	NA	NA	NA	NA	NA	NA	NA	23	11	14	47	65	19
Shin (2003)	26	101	89	65	299	384	NA	NA	NA	NA	NA	NA	201	28	1	675	112	5
Shin (2011)	60	291	283	32	108	103	NA	NA	NA	NA	NA	NA	534	91	7	199	38	0
Sicinschi (2006)	51	90	40	98	176	95	NA	NA	NA	NA	NA	NA	NA	NA	NA	NA	NA	NA
Sugimoto (2007)	8	54	43	10	70	88	6	57	42	9	73	86	78	26	0	134	34	0
Sun (2013)	31	163	162	33	141	191	NA	NA	NA	NA	NA	NA	NA	NA	NA	NA	NA	NA
Talseth (2007)	62	51	4	50	45	4	NA	NA	NA	NA	NA	NA	36	61	21	33	50	17
Tseng (2006)	31	84	91	27	147	169	NA	NA	NA	NA	NA	NA	NA	NA	NA	NA	NA	NA
Tsilidis (2009)	123	71	9	213	131	17	NA	NA	NA	NA	NA	NA	69	101	35	98	187	87
Vishnoi (2007)	NA	NA	NA	NA	NA	NA	43	61	20	70	93	37	NA	NA	NA	NA	NA	NA
Wu (2003)	27	105	88	20	83	127	27	105	88	20	83	127	195	23	2	217	11	2
Zambon (2005)	70	42	17	353	245	46	70	42	17	353	245	46	48	56	25	232	326	86
Zeng (2012)	15	77	59	7	66	80	11	80	60	10	65	78	27	60	64	48	53	52
Zhou (2011)	NA	NA	NA	NA	NA	NA	NA	NA	NA	NA	NA	NA	29	62	59	52	53	45

Abbreciations: NA, not available.

Supplementary Table S2. The overall and subgroup analyses of -592C>A and -819C>T in IL-10 gene with digesitve cancer risk

Groups	Studies	Allelic model		Homozygous genotypic model		Dominant model	
		OR; 95% CI; P	*I*-squared	OR; 95% CI; P	*I-squared*	OR; 95% CI; P	*I-squared*
**IL-10 gene -592C>A**							
**Overall**	41	0.988; 0.922-1.058; 0.723	55.9%	0.978; 0.842-1.136; 0.774	45.1%	1.049; 0.943-1.168; 0.379	50.0%
**Ethnicity**							
Caucasian	11	0.993; 0.884-1.114; 0.900	36.3%	0.929; 0.659-1.310; 0.675	45.3%	0.998; 0.882-1.128; 0.969	18.0%
East Asian	23	0.953; 0.873-1.040; 0.280	57.6%	0.962; 0.799-1.160; 0.687	50.6%	1.015; 0.858-1.202; 0.859	49.2%
Latinos	4	1.189; 0.884-1.114; 0.375	78.5%	1.113; 0.577-2.148; 0.750	45.3%	1.296; 0.781-2.152; 0.316	77.5%
Mixed	2	1.043; 0.698-1.558; 0.839	67.7%	1.393; 0.676-2.869; 0.369	43.5%	0.967; 0.645-1.450; 0.872	53.1%
Indian	1	0.990; 0.644-1.522; 0.962	-	0.545; 0.133-2.235; 0.400	-	1.835; 0.822-4.098; 0.139	-
**Sample size**							
< 500 subjects	19	0.944; 0.816-1.091; 0.434	64.7%	0.828; 0.619-1.107; 0.202	47.7%	1.030; 0.827-1.283; 0.791	57.9%
>= 500 subjects	22	1.008; 0.940-1.082; 0.818	45.3%	1.053; 0.890-1.246; 0.550	37.3%	1.046; 0.931-1.175; 0.452	43.5%
**Cancer type**							
Esophageal cancer	3	0.908; 0.772-1.067; 0.239	0.0%	0.986; 0.682-1.426; 0.939	0.0%	0.941; 0.727-1.218; 0.645	0.0%
Gastric cancer	21	0.980; 0.884-1.086; 0.699	62.3%	0.979; 0.797-1.202; 0.837	45.9%	1.031; 0.884-1.202; 0.697	50.6%
Colerectal cancer	7	1.036; 0.893-1.203; 0.640	30.8%	0.953; 0.584-1.553; 0.846	41.0%	1.061; 0.902-1.248; 0.473	16.8%
Hepatocelluar carcinoma	7	0.886; 0.771-1.018; 0.087	40.3%	0.751; 0.476-1.183; 0.217	63.3%	0.944; 0.653-1.364; 0.758	61.5%
Biliary tract cancer	3	1.296; 1.029-1.633; 0.028	45.9%	1.478; 0.842-1.136; 0.108	52.6%	1.746; 0.831-3.670; 0.141	82.2%
**GC anatomical type**							
Cardia type	6	0.908; 0.755-1.093; 0.308	0.0%	0.859; 0.548-1.348; 0.509	0.0%	0.896; 0.710-1.130; 0.354	0.0%
Non-cardia type	9	1.050; 0.901-1.223; 0.531	56.9%	1.154; 0.789-1.688; 0.459	58.3%	1.068; 0.926-1.231; 0.365	0.0%
**GC histology type**							
Intestinal type	7	0.938; 0.795-1.106; 0.446	47.9%	0.913; 0.647-1.288; 0.604	30.2%	0.978; 0.797-1.199; 0.828	0.0%
Diffuse type	7	0.978; 0.911-1.137; 0.770	24.0%	1.026; 0.771-1.366; 0.858	0.0%	1.072; 0.858-1.339; 0.542	0.0%
**Matched**							
NA	11	1.059; 0.888-1.262; 0.523	73.7%	1.045; 0.702-1.555; 0.828	65.3%	1.083; 0.837-1.400; 0.546	71.6%
YES	26	0.980; 0.908-1.058; 0.610	48.4%	0.997; 0.850-1.170; 0.974	36.4%	1.032; 0.918-1.162; 0.595	38.8%
NO	4	0.882; 0.737-1.057; 0.174	0.0%	0.603; 0.325-1.121; 0.110	0.0%	1.010; 0.682-1.495; 0.962	0.0%
**Control source**							
Population	23	1.026; 0.930-1.131; 0.614	57.3%	0.999; 0.809-1.234; 0.995	40.6%	1.084; 0.931-1.261; 0.298	60.9%
Hospital	18	0.946; 0.859-1.041; 0.253	53.8%	0.956; 0.768-1.191; 0.689	52.0%	1.010; 0.871-1.172; 0.893	29.2%
**Study design**							
Retrospective	31	1.004; 0.921-1.094; 0.930	62.6%	0.990; 0.832-1.179; 0.912	49.8%	1.100; 0.956-1.266; 0.181	54.2%
Prospective	10	0.947; 0.863-1.039; 0.249	4.5%	0.937; 0.698-1.258; 0.664	25.0%	0.932; 0.828-1.048; 0.241	0.0%
**IL-10 gene -819C>T**							
**Overall**	24	0.935; 0.849-1.030; 0.173	53.3%	0.873; 0.685-1.114; 0.275	54.1%	0.892; 0.741-1.073; 0.224	60.2%
**Ethnicity**							
Caucasian	5	1.041; 0.778-1.392; 0.788	72.8%	1.084; 0.477-2.464; 0.847	74.0%	1.028; 0.766-1.379; 0.856	59.8%
East Asian	15	0.899; 0.798-1.013; 0.081	56.2%	0.793; 0.598-1.050; 0.105	53.5%	0.793; 0.590-1.067; 0.125	66.0%
Latinos	1	0.727; 0.383-1.380; 0.330	-	0.960; 0.191-4.826;0.960	-	0.533; 0.231-1.232; 0.141	-
Mixed	1	1.079; 0.795-1.464; 0.625	-	1.512; 0.724-3.157; 0.271	-	0.987; 0.674-1.444; 0.946	-
Indian	2	0.970; 0.749-1.255; 0.815	0.0%	0.807; 0.443-1.471; 0.484	0.0%	1.243; 0.716-2.159; 0.440	35.9%
**Sample size**							
< 500 subjects	15	0.905; 0.793-1.032; 0.137	48.1%	0.862; 0.643-1.157; 0.322	33.4%	0.987; 0.818-1.192; 0.895	25.7%
>= 500 subjects	9	0.974; 0.849-1.030; 0.721	60.8%	0.880; 0.574-1.350; 0.558	74.7%	0.773; 0.544-1.100; 0.152	78.6%
**Cancer type**							
Esophageal cancer	1	0.986; 0.724-1.343; 0.928	-	0.899; 0.478-1.691; 0.740	-	0.857; 0.472-1.555; 0.611	-
Gastric cancer	11	0.866; 0.771-0.973; 0.016	35.1%	0.880; 0.668-1.159; 0.363	32.8%	0.870; 0.744-1.017; 0.080	0.0%
Colerectal cancer	3	1.200; 0.808-1.782; 0.365	72.0%	1.389; 0.339-5.693; 0.648	77.5%	1.229; 0.836-1.807; 0.293	56.1%
Hepatocellular carcinoma	5	0.987; 0.846-1.151; 0.867	0.0%	0.774; 0.429-1.396; 0.394	0.0%	1.027; 0.689-1.530; 0.896	13.1%
Biliary tract cancer	4	0.908; 0.648-1.272; 0.575	79.9%	0.736; 0.365-1.486; 0.393	81.7%	0.643; 0.283-1.462; 0.292	89.7%
**GC anatomical type**							
Cardia type	4	0.959; 0.750-1.226; 0.738	0.0%	1.158; 0.662-2.026; 0.606	0.0%	0.889; 0.609-1.299; 0.544	0.0%
Non-cardia type	5	0.914; 0.779-1.071; 0.264	25.6%	0.925; 0.549-1.558; 0.770	52.7%	0.952; 0.775-1.168; 0.635	0.0%
**GC histology type**							
Intestinal type	3	0.847; 0.705-1.018; 0.076	0.0%	0.831; 0.540-1.279; 0.400	0.0%	0.947; 0.666-1.348; 0.763	0.0%
Diffuse type	3	0.761; 0.517-1.118; 0.164	47.4%	0.952; 0.570-1.590; 0.851	0.0%	0.888; 0.548-1.438; 0.628	12.8%
**Matched**							
NA	3	1.239; 0.852-1.804; 0.262	69.5%	1.516; 0.386-5.956; 0.551	77.8%	1.245; 0.861-1.801; 0.244	52.2%
YES	4	0.909; 0.816-1.013; 0.085	51.7%	0.832; 0.655-1.059; 0.135	47.0%	0.806; 0.643-1.010; 0.061	63.0%
NO	17	0.880; 0.737-1.050; 0.156	0.0%	0.641; 0.338-1.217; 0.174	0.0%	0.974; 0.654-1.450; 0.896	7.2%
**Control source**							
Population	13	0.921; 0.794-1.067; 0.271	62.6%	0.791; 0.538-1.162; 0.232	65.0%	0.823; 0.607-1.115; 0.208	74.6%
Hospital	11	0.952; 0.839-1.081; 0.448	41.4%	1.002; 0.758-1.324; 0.989	27.5%	1.003; 0.847-1.188; 0.975	0.0%
**Study design**							
Retrospective	19	0.949; 0.843-1.069; 0.388	60.9%	0.895; 0.675-1.187; 0.442	59.9%	0.913; 0.723-1.153; 0.443	66.6%
Prospective	5	0.880; 0.766-1.010; 0.069	0.0%	0.758; 0.500-1.148; 0.191	0.0%	0.802; 0.646-0.996; 0.046	0.0%

Abbreviations: NA, not available; OR, odds ratio; 95% CI, 95% confidence interal; *I*-squared, inconsistency index.

Supplementary Table S3. Changes of circulating IL-10 level across genotypes of three examined variants in IL-10 gene

IL-10 gene -592C>A	Ethnicity	Status	Sample size	CC genotype		CA genotype		AA genotype	
Authoryear				Number	IL-10 level (pg/ml)	Number	IL-10 level (pg/ml)	Number	IL-10 level (pg/ml)
Hohaus (2009)	Caucasian	Cases	95	NA	NA	85	29.20	10	53.10
Jin (2013)	East Asian	Cases	180	96	10.1	84	13.20	NA	NA
Munro (2003)	Caucasian	Cases	25	15	106	10	35.90	NA	NA
Roh (2002) a	East Asian	Controls	179	15	3.93	77	3.20	87	13.20
Roh (2002) b	East Asian	Cases	144	11	2.55	56	4.22	77	3.17
Saxena (2014) a	Indian	Controls	145	36	51.08	87	70.22	32	73.38
Saxena (2014) b	Indian	Cases	62	5	36.69	52	80.58	5	0.00
Saxena (2014) c	Indian	Cases	183	20	71.94	156	78.85	7	0.00
IL-10 gene -819C>T	Ethnicity	Status	Sample size	CC genotype		CT genotype		TT genotype	
Jin (2013)	East Asian	Cases	180	99	9.60	81	12.80	NA	NA
Roh (2002) a	East Asian	Controls	179	15	3.93	77	3.20	87	13.20
Roh (2002) b	East Asian	Cases	144	11	2.55	56	4.22	77	3.17
Sakamoto (2014)	East Asian	Cases	86	NA	NA	49	9.60	37	3.80
Saxena (2014) a	Indian	Controls	145	36	51.08	87	70.22	32	73.38
Saxena (2014) b	Indian	Cases	62	5	36.69	52	80.58	5	0.00
Saxena (2014) c	Indian	Cases	183	20	71.94	156	78.85	7	0.00
Sun (2010)	East Asian	Cases	116	7	59.71	NA	NA	50	54.18
IL-10 gene -1082A>G	Ethnicity	Status	Sample size	AA genotype		AG genotype		GG genotype	
Hohaus (2009)	Caucasian	Cases	95	NA	NA	87	29.20	8	56.20
Jin (2013)	East Asian	Cases	180	68	10.70	112	12.60	NA	NA
Munro (2003)	Caucasian	Cases	26	3	43.00	11	67.80	12	91.70
Sun (2010)	East Asian	Cases	116	97	50.13	19	94.31	0	0.00
